# Gas Plasma Exposure of Glioblastoma Is Cytotoxic and Immunomodulatory in Patient-Derived GBM Tissue

**DOI:** 10.3390/cancers14030813

**Published:** 2022-02-05

**Authors:** Sander Bekeschus, Mikael Ispirjan, Eric Freund, Frederik Kinnen, Juliane Moritz, Fariba Saadati, Jacqueline Eckroth, Debora Singer, Matthias B. Stope, Kristian Wende, Christoph A. Ritter, Henry W. S. Schroeder, Sascha Marx

**Affiliations:** 1 ZIK *plasmatis*, Leibniz Institute for Plasma Science and Technology (INP), Felix-Hausdorff-Str. 2, 17489 Greifswald, Germany; mikael.ispirjan@stud.uni-greifswald.de (M.I.); eric.freund@inp-greifswald.de (E.F.); fk133993@uni-greifswald.de (F.K.); juliane.moritz@inp-greifswald.de (J.M.); fariba.saadati@inp-greifswald.de (F.S.); jacqueline.eckroth@inp-greifswald.de (J.E.); debora.singer@inp-greifswald.de (D.S.); kristian.wende@inp-greifswald.de (K.W.); 2 Department of Neurosurgery, Greifswald University Medical Center, Ferdinand-Sauerbruch-Str., 17475 Greifswald, Germany; henry.schroeder@uni-greifswald.de (H.W.S.S.); sascha.marx@uni-greifswald.de (S.M.); 3 Department of General, Visceral, Thoracic, and Vascular Surgery, Greifswald University Medical Center, Ferdinand-Sauerbruch-Str., 17475 Greifswald, Germany; 4 Clinic and Policlinic for Dermatology and Venerology, Rostock University Medical Center, Strempelstr. 13, 18057 Rostock, Germany; 5 Department of Gynecology and Gynecological Oncology, Bonn University Medical Center, Venusberg-Campus 1, 53127 Bonn, Germany; matthias.Stope@ukbonn.de; 6 Department of Clinical Pharmaceutics, University of Greifswald, Felix-Hausdorff-Str. 1, 17489 Greifswald, Germany; ritter@uni-greifswald.de; 7 Department of Cancer Immunology and Virology, Dana-Farber Cancer Institute, Harvard Medical School, Boston, MA 02215, USA

**Keywords:** brain tumor, chemokines, cytokines, cold physical plasma, reactive oxygen species

## Abstract

**Simple Summary:**

Despite treatment advances, glioblastoma multiforme (GBM) remains an often-fatal disease, motivating novel therapeutic avenues. Gas plasma is a technology that has been recently employed in preclinical oncology research and acts primarily via reactive oxygen-species-induced cell death. In addition, the modulation of immune processes and inflammation have been ascribed to gas plasma exposure. This is the first study that extends those observations from in vitro investigations to a set of 16 patient-derived GBM tumor biopsies analyzed after gas plasma treatment ex vivo. Besides cell culture results showing cell cycle arrest and apoptosis induction, an immunomodulatory potential was identified for gas plasma exposure in vitro and cultured GBM tissues. The proapoptotic action shown in this study might be an important step forward to the first clinical observational studies on the future discovery of gas plasma technology’s potential in neurosurgery and neuro-oncology.

**Abstract:**

Glioblastoma multiforme (GBM) is the most common primary malignant adult brain tumor. Therapeutic options for glioblastoma are maximal surgical resection, chemotherapy, and radiotherapy. Therapy resistance and tumor recurrence demand, however, new strategies. Several experimental studies have suggested gas plasma technology, a partially ionized gas that generates a potent mixture of reactive oxygen species (ROS), as a future complement to the existing treatment arsenal. However, aspects such as immunomodulation, inflammatory consequences, and feasibility studies using GBM tissue have not been addressed so far. In vitro, gas plasma generated ROS that oxidized cells and led to a treatment time-dependent metabolic activity decline and G2 cell cycle arrest. In addition, peripheral blood-derived monocytes were co-cultured with glioblastoma cells, and immunomodulatory surface expression markers and cytokine release were screened. Gas plasma treatment of either cell type, for instance, decreased the expression of the M2-macrophage marker CD163 and the tolerogenic molecule SIGLEC1 (CD169). In patient-derived GBM tissue samples exposed to the plasma jet kINPen ex vivo, apoptosis was significantly increased. Quantitative chemokine/cytokine release screening revealed gas plasma exposure to significantly decrease 5 out of 11 tested chemokines and cytokines, namely IL-6, TGF-β, sTREM-2, b-NGF, and TNF-α involved in GBM apoptosis and immunomodulation. In summary, the immuno-modulatory and proapoptotic action shown in this study might be an important step forward to first clinical observational studies on the future discovery of gas plasma technology’s potential in neurosurgery and neuro-oncology especially in putative adjuvant or combinatory GBM treatment settings.

## 1. Introduction

Glioblastoma multiforme (GBM) is characterized as a WHO-grade-IV tumor and, therefore, a highly aggressive primary brain tumor. GBMs are among the most malignant brain tumors and have a poor outcome with a median survival of only 14 months. The 5-year overall survival is meager, with an average of 4–6% [1]. The current standard of care is maximal surgical resection, adjuvant chemotherapy, and radiotherapy. GBM is typically characterized by strong resistance to apoptosis by radiation and chemotherapeutic treatment regimens [2]. Due to the limited success of treatment, the development of new therapeutic strategies is indispensable.

In recent years, gas plasma technology has been heavily investigated as a novel anti-cancer agent [3]. It became evident that the local deposition of a plethora of reactive oxygen and nitrogen species (ROS/RNS) is the principal mechanism by which gas plasmas mediate biological effects, including apoptosis in cancer cells [4]. Intriguingly, certified medical plasma technology, such as the kINPen as the only example of an approved plasma jet, is operated at body temperature [5], sparing vital tissue from thermal damage. At the same time, plasmas jets can be operated by surgeons like a knife, with a high precision achievable for the tissue to be targeted. Moreover, the jet plasma seamlessly enters microcavities that would not be penetrated with flat-surface plasma sources. This array of reasons motivated us to provide a proof-of-concept study on the plasma jet kINPen for targeting glioblastoma. This was supported by the fact that several in vitro studies have investigated the effects of direct gas plasma exposure and treatment with plasma-treated liquids in different GBM cell lines in vitro [6,7,8,9] and—non-orthotopically—in vivo [10,11,12]. Some studies have also investigated the combination of gas plasma with drug treatment [13,14,15].

In solid cancers in general, and GBM specifically, not only the tumor cells themselves but also their surrounding are therapeutic targets. The composition of this micro milieu, called the tumor microenvironment (TME), has been found to dictate the outcome of many types of therapies in GBM and other cancers [16,17]. With the arrival of cancer immunotherapies entering most oncological treatment schemes, there is also an increasing understanding of the critical role of tumor-infiltrating leukocytes and median survival, as these leukocytes may perform direct tumor-toxic or promoting action by cell–cell-interactions or the release of soluble mediators, such as chemokines, cytokines, and growth factors [18,19,20]. GBM in particular is known to acquire therapy resistance quickly. Moreover, GBM is often heavily infiltrated with tumor-associated macrophages (TAM) and microglial cells that promote tumor growth [21]. To this end, it is not only important to learn how a therapeutic approach affects tumor cells but also how it affects cells of the immune system as well as how therapeutically targeted tumor cells signal into the TME and vice versa [16,22].

The current study aimed to provide a proof-of-concept preclinical study on employing a gas plasma jet-based anti-GBM approach, taking into account the many facets of such a strategy, among them (i) showing the principal ROS-generating nature, (ii) providing a basic understanding of toxicity based on cell cycle arrest, (iii) deciphering the immunomodulatory features by using plasma co-culture models, and (iv) elucidating the toxicity and effects on chemokine and cytokine as well as growth factor secretion following gas plasma exposure to primary patient-derived glioblastoma tissue biopsies ex vivo. By including 16 diseased individuals in our study, this is the first time the proapoptotic nature of gas plasma treatment was shown in human glioblastoma samples. These results could be helpful to target surgical margins after GBM excision to reduce disease recurrences.

## 2. Materials and Methods

### 2.1. Cell Culture

Non-malignant HaCaT keratinocytes (ATCC: PCS-200-011) and malignant T98G (ATCC: CRL-1690) and U87 (ATCC: HTB-14) glioblastoma cell lines were cultured up to passage number 15 from the original stock under standard conditions at 37 °C, 5% CO_2_, and 95% humidity in a sterilizable cell culture incubator (Binder, Stuttgart, Germany). Cells were maintained in a fully supplemented Roswell Park Memorial Institute (RPMI) 1640 cell culture medium supplemented with fetal bovine serum (10%), glutamine (2%), and penicillin–streptomycin (1%). Cells received fresh medium twice to thrice per week and were sub-passaged usually once a week using trypsinization.

### 2.2. Monocyte Isolation

Peripheral blood was obtained from venipuncture from four donors upon informed consent. Peripheral blood mononuclear cells (PBMC) were obtained as described [23]. Briefly, blood was layered above a high-density glucose solution, followed by centrifugation without a break, PBMC collection, and hypotonic lysis of contaminating red cells. Subsequently, CD14^+^ monocytes were isolated by magnetic bead purification based on a negative selection kit (Miltenyi Biotec, Teterow, Germany).

### 2.3. Glioblastoma Multiforme (GBM) Tissue Biopsies

Brain tumor tissue biopsies were collected from 16 patients (4 female and 12 male, mean age 67 (range: 41–85 years)) who were radiologically suspected to have glioblastoma multiforme. The diagnoses were later confirmed by the Institute of Pathology in Greifswald, Germany. The tumor samples were removed intraoperatively with tumor-grasping forceps. Depending on operability, several biopsies of different areas were taken in the surgical theater and transported in 0.9% physiological sodium chloride solution to the laboratory. One or several punch biopsies (diameter: 5 mm) were generated from each tissue sample. This way, several technical replicates could be treated and analyzed from each sample type. Sample culturing was done by transfer into 24-well plates containing cell culture medium and incubated under standard culture conditions. Twenty-four hours later, supernatants were collected and stored at −80 °C. Tissues were embedded in Tissue Tek Cryomold (Sakura Finetek, Staufen, Germany) in disposable base molds (Thermo Fisher Scientific, Bremen, Germany), snap-frozen in liquid nitrogen, wrapped in aluminum foil, and stored at −80 °C.

### 2.4. Gas Plasma Treatment

The atmospheric pressure argon plasma jet kINPen was utilized for gas plasma jet exposure. The technical properties of the device have been extensively described previously [24]. The jet was operated using 3 standard liters per minute of argon gas (purity: 99.999%; Air Liquide, Bremen, Germany). In some in vitro experiments, the argon gas was humidified before being fed into the jet, as described before [25]. As an additional iteration in the in vitro studies, a surface dielectric barrier discharge (DBD) was utilized as outlined previously [26]. The kINPen operated with dry or humid (wet) argon or the DBD was placed above a 60 mm dish containing 5 mL of fully supplemented cell culture medium, operated for the given exposure time, which was subsequently transferred to the cells. For gas plasma exposure in the co-culture experiments, only 30 s of kINPen treatment (with dry argon) of either monocytes or tumor cells (2 × 10^4^ per well of a 24-well plate) was performed. For gas plasma exposure of the patient-derived GBM samples, the tissue biopsies were placed into small tips that were subsequently added to the wells of a 96-well plate. This way, a computer-programmed *xyz* table (CNC, Prenzlau, Germany) with the kINPen attached was able to gas plasma-treat several samples at a similar height and location (central to the tissue sample) for 120 s. The treatment was performed within 2 h postsurgery in the laboratory before a 18 h incubation period at 37 °C. All control samples remained untreated.

### 2.5. ROS and Metabolic Activity Analysis

For in vitro experiments and results, hydrogen peroxide (H_2_O_2_) is a primary biological mediator of gas plasma effects in liquids in contact with cells [27]. H_2_O_2_ was quantified in the gas plasma-treated cell culture medium (without cells) within 30 min after treatment as described in the manufacturer’s protocol of the Amplex Red kit (Thermo Fisher, Bremen, Germany). Subsequently, the plate was added to a bandpass-filter-based microplate reader (F200; Tecan, Männedorf, Schweiz), and the wells’ fluorescence was quantified with λ_ex_ 560 nm and λ_em_ 590 nm. Prior to assessing intracellular ROS increase, 1 × 10^4^ HaCaT or T98G cells were added to each well of a 96-well plate and were allowed to adhere overnight. The next day, the medium was aspirated, and the cells were stained with H_2_-DCF-DA according to the vendor’s instructions (Thermo Fisher, Bremen, Germany). Fresh untreated or gas plasma-treated medium was added, followed by quantification in the microplate reader with λ_ex_ 485 nm and λ_em_ 525 nm 30 min later. For analyzing metabolic activity, 2.5 × 10^3^ cells were seeded in 96-well plates and allowed to adhere overnight. The next day, the medium was replaced with fresh untreated or gas plasma-treated medium. Then, 68 h later, resazurin (final concentration: 100 µM; Thermo Fisher, Bremen, Germany) was added, and the cells were incubated for another 4 h at 37 °C. Then, the 96-well plate was transferred to the microplate reader, and fluorescence was quantified at λ_ex_ 560 nm and λ_em_ 590 nm.

### 2.6. Cell Cycle Analysis

For cell cycle analysis, 5 × 10^4^ cells per well of a 24-well plate were exposed to gas plasma-treated medium. Twenty-four hours later, cells were collected, washed, fixed, and permeabilized with ice-cold ethanol, incubated with RNAse, and stained with propidium iodide (final concentration: 10 µg/mL; ThermoFisher, Bremen, Germany). After washing, samples were acquired using flow cytometry (Gallios; Beckman Coulter, Krefeld, Germany). Sample analysis was carried out using Kaluza 2.1 software (Beckman Coulter, Krefeld, Germany), and the integrated FOX algorithm was used to retrieve the percentage of cells in the G1, S, and G2 phases from mathematical modeling of doublet-cleared input gates.

### 2.7. Co-Culture and Multicolor Surface Marker Flow Cytometry

Co-cultures of human peripheral blood-derived monocytes and T87MG GBM cells were set up in 24-well plates with 2 × 10^4^ cells of each entity in 500 µL of fully supplemented cell culture medium. Specifically, 2 × 10^4^ cells of one entity was added to the wells, followed by gas plasma treatment (30 s) of this suspension. Four hours later, the complementary cell (U87 GBM cells to gas plasma-treated monocytes; monocytes to gas plasma-treated GBM cells) type was added in 500 µL of cell culture medium. After co-culture incubation for at least 48 h, the supernatant was collected and stored for later longitudinal analysis, and the cells were harvested using trypsin. After washing, the cells were incubated with different fluorescently labeled monoclonal antibody master mixes that targeted (some of) the following molecules: cluster of differentiation (CD)11, CD14, CD45, CD55, CD97, CD162, CD169, CD204, CD276, HLA-ABC, and HLA-DR. Isotype controls were IgG1, IgG2a, IgG2b, and REA antibodies. The antibodies were labeled with different fluorophores, such as phycoerythrin (PE), peridinin–chlorophyll–protein Complex (PerCP), Fluorescein-5-isothiocyanate (FITC), allophycocyanin (APC), PE-Vio770, PerCP-Vio700, and VioBright-FITC. A Tandem signal enhancer in running buffer was added to all samples. After staining, samples were washed in running buffer, resuspended in running buffer, and acquired using flow cytometry (MACS-Quant10). 4′,6-Diamidin-2-phenylindol (DAPI) was used to label terminally dead cells. All material was supplied by Miltenyi Biotec, Bergisch Gladbach, Germany.

### 2.8. Multiplex Chemokine and Cytokine Analysis

Multiplex chemokine, cytokine, and growth factor quantification was performed using a bead-based assay (BioLegend, Amsterdam, The Netherlands) according to the vendor’s instructions. Briefly, supernatants of the in vitro co-culture assays and ex vivo GBM tissue cultures were incubated with beads, and mean fluorescence intensities (MFI) of each bead population (representing a single analyte) were determined using flow cytometry (CytoFLEX S; Beckman Coulter, Krefeld, Germany). Total analyte concentrations were calculated in picogram per milliliter against a known standard using 5-log fitting with dedicated VigeneTech software. Statistical analysis was performed by parametric paired *t*-tests for each analyte and donor.

### 2.9. Tissue Sections and Immunofluorescence Staining, Imaging, and Analysis

The tissue samples were sectioned vertically (thickness: 5 µm) 90° to the gas plasma-treated side (facing up) on Superfrost plus slides (Thermo Fisher, Bremen, Germany) and stored at −80 °C until further staining. Prior to staining, the sections were fixed in 4% paraformaldehyde (Carl Roth, Karlsruhe, Germany) and washed three times for five minutes in phosphate-buffered saline (PBS). Cell membrane permeabilization was performed for two minutes using 0.25% Triton X-100 (Carl Roth, Karlsruhe, Germany) followed by three washing periods of five minutes in PBS. The TUNEL kit (Roche Diagnostics, Basel, Switzerland) was used for apoptosis detection as per the manufacturer’s instructions. The slides were incubated for one hour at 37 °C in a humidified chamber. The sections were washed three times in PBS followed by DAPI addition to counterstain nuclei. The slides were mounted with Fluoromount Aqueous Mounting Medium (Sigma-Aldrich, Taufkirchen, Germany). Imaging and analysis were performed using a high-content imaging device (Operetta CLS; PerkinElmer, Hamburg, Germany) and its associated software (Harmony 4.9; PerkinElmer, Hamburg, Germany). Quantification was based on algorithm-driven object segmentation.

### 2.10. Statistical Analysis

Prism 9.3 (GraphPad Software, San Diego, CA, USA) was used for graphing and statistical analysis. The details of statistical analysis are given in the figure legends. Level of significance is indicated as follows: α = 0.05 (*), α = 0.01 (**), and α = 0.001 (***).

## 3. Results

### 3.1. Metabolic Activity Decline and Cell Cycle Rest of Glioblastoma Monocultures In Vitro

**Figure 1:**
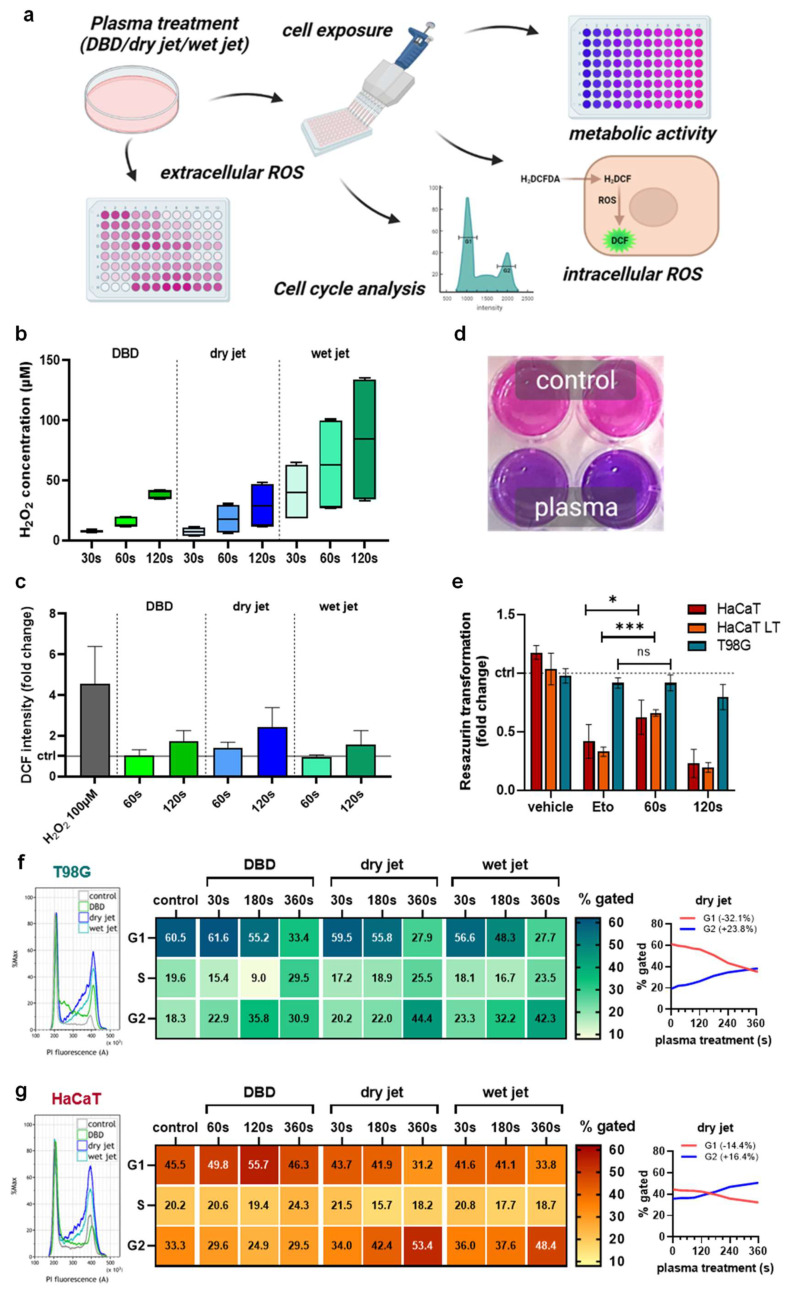
Metabolic activity decline and cell cycle rest of glioblastoma monocultures in vitro. (**a**) study scheme; (**b**) H_2_O_2_ generation by the DBD and the jet operated with dry and humid (wet) argon gas; (**c**) cellular oxidation as indicated by DCF fluorescence upon exposure to plasma-treated media; (**d**,**e**) representative macroscopic image of the resazurin assay (**d**) and metabolic activity quantified and normalized (**e**) against each untreated control for HaCaT keratinocytes, HaCaTs with a history of plasma exposure and passaging (LT) and malignant T98G GBM cells; (**f**,**g**) representative flow cytometry overlay histograms of propidium iodide (PI) fluorescence (left), quantification of cell cycle phases using mathematically modeling (middle), and spaghetti plots of the same data visualizing G1 to G2 ratios for T98G (**f**) and HaCaT (**g**) cells. Data are representative of boxplot or mean and standard error of three experiments; statistical analysis was performed using unpaired, two-tailed *t*-test with *p* < 0.05 (*) and *p* < 0.001 (***) differing significantly or non-significantly (ns).

To analyze the efficacy of gas plasma technology in vitro, different plasma setups were tested to treat cells (Figure 1a). The idea was to show that different plasma source approaches may be able to perform a similar task. Both the DBD and the jet-generated H_2_O_2_ as a representative long-lived oxidant in an exposure-time-dependent fashion (Figure 1b). The plasma jet’s chemistry can be optimized by, e.g., changing the feed gas composition. Accordingly, humidified argon gas extended the H_2_O_2_ generation compared to the dry condition. The oxidants introduced by the plasma sources oxidized the treated cells, as evidenced by increased DCF intensities (Figure 1c). Next, the dry jet’s ability to reduce the cells’ metabolic activity (Figure 1d) was used. HaCaT keratinocytes were exposed either once or several times to the plasma-treated medium (LT) with passaging in between serving as a non-malignant control compared to T98G GBM cells. The apoptosis-induced etoposide (Eto, positive control) was effective in HaCaT but only to a minor extent in the malignant T98G cells (Figure 1e). Interestingly, the 60 s gas plasma condition conferred only minor toxicity in GBM cells as well, while it was significantly more toxic in HaCaTs. Longer plasma exposure times (120 s) were toxic in both cell types. To study the consequences of gas plasma-generated ROS/RNS on proliferative capacity, cell cycle analysis was performed in T98G (Figure 1f) and HaCaT keratinocytes (Figure 1g). For all plasma treatment approaches, a prominent G2 arrest was observed (left histograms). Mathematical modeling (Michael H. Fox algorithm) of cell cycle phases and quantification supported this notion (middle). The largest percentage of cells observed in the G2 phase was found for the longest jet treatment (dry) in both cell lines. Spaghetti plots (right) illustrate the conversion of a G1-phase dominated into a G2-phase dominated cellular phenotype. In terms of absolute changes from the original state, this was much more pronounced in GBM cells (G1: −32.1%, G2: +23.8%) than in HaCaTs (G1: −14.4%, G2: +16.4%).

### 3.2. Immunomodulation in Gas Plasma-Treated Monocyte–GBM Co-Cultures

**Figure 2:**
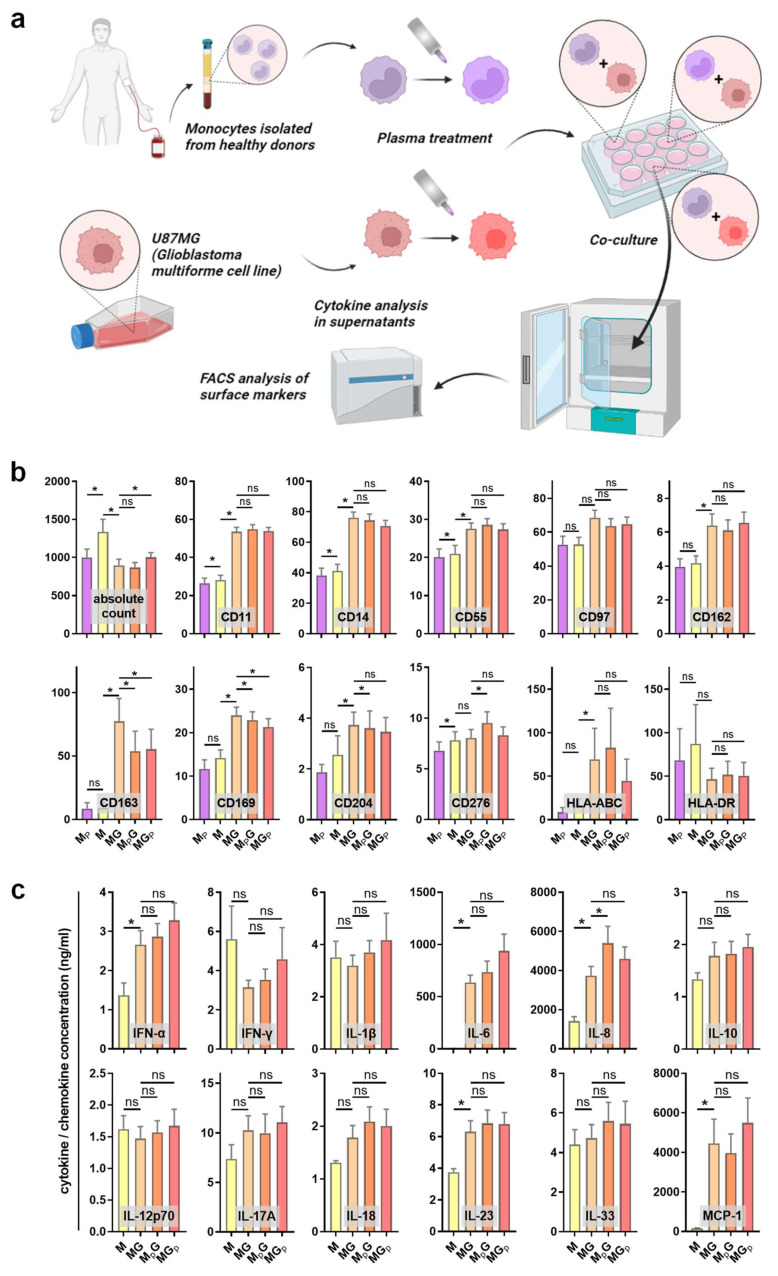
Monocyte surface marker expression and cytokine release profiles in GBM cultures. (**a**) Study protocol; (**b**) absolute viable monocyte counts and median fluorescence intensities of monocytes (M), gas plasma-treated monocytes (M_P_), monocyte–GBM co-cultures (MG), gas plasma-treated monocytes co-cultured with GBM (M_P_G), and gas plasma-treated GBM cells co-cultured with monocytes (MG_P_) as determined using multicolor flow cytometry; (**c**) cytokine, chemokine, and growth factor release quantification in monocyte monoculture and co-culture supernatants. Data are mean and standard error of monocytes from four donors; statistical analysis was performed using two-tailed Wilcoxon rank test with *p* < 0.05 (*) differing significantly or non-significantly (ns).

To analyze the immunomodulatory effect of gas plasma treatment, peripheral blood-derived monocytes were isolated and co-cultured with GBM cells. Either the monocytes or the GBM cells were exposed to gas plasma, followed by the addition of the complementary cell type afterward. The surface marker expression of monocytes was investigated using multicolor flow cytometry, and the co-cultures’ supernatants were quantitatively assessed for chemokine, cytokine, and growth factor release (Figure 2a). Monocultures of monocytes served as control. Sufficient numbers of viable monocytes were measured in the different conditions to assess their surface marker profiles (Figure 2b). Gas plasma exposure of monocytes (M_P_) compared to monocytes alone (M) only had a minor effect on the cells. A subtle but significant decrease was observed for CD11 and CD14, both being macrophage differentiation markers (but usually regulated at much greater amplitude), as well as CD55 being an activation marker and CD276 (B7-H3), a prominent immunosuppressant, apart from CD169. Significant and somewhat greater changes were observed between the monoculture and co-culture of monocytes with GBM cells, as seen by a significant increase in CD11, CD14, CD55, CD97, CD162, CD163, CD169, and CD204. CD162 (PSGL-1) is a pro-atherogenic marker as it binds platelets, CD163 is a marker of tumor-supporting macrophages, CD169 defines tolerogenic (anti-inflammatory) monocytes binding to red cells, CD97 is an EGF-TM7 receptor, and pattern-recognition-receptor CD204 engagement is pro-inflammatory. The co-culture of gas plasma-treated monocytes with GBM cells gave a modest but significant decrease in CD14, CD97, CD163, and CD169 expression, while CD276 expression was significantly increased. In case of gas plasma-treated GBM cells subsequently co-cultured with monocytes, a significant decrease in CD14, CD163, and CD169 was observed on monocytes. These results indicate functional changes of monocytes with gas plasma exposure, albeit the overall amplitudes of changes were moderate. However, a monovalent response type could not be identified, as both activation and suppression markers were regulated in favor and disfavor of anti-tumor activity simultaneously. Next, the release of chemokines, cytokines, and growth factors was quantified (Figure 2c). Again, the co-culture was markedly different from monocyte monocultures, especially IL-6 (Interleukin), IL-8, IL-23, and MCP-1 (monocyte chemoattractant protein 1). Our study could not establish whether these analytes were a product of the tumor cells or the monocytes affected by the tumor cells due to the lack of GBM monocultures. In co-cultures, gas plasma exposure to monocytes significantly increased IL-6 and IL-8 levels, while the levels of all other released factors did not change significantly. IL-8 has a great degree of pleiotropy in health and disease, including cancer. In summary, gas plasma treatment was perceived by the monocytes and affected their phenotype and the inflammatory mediator release profile in co-cultures with GBM cells.

### 3.3. Analysis of Gas Plasma-Treated Patient-Derived GBM Tissue

**Figure 3:**
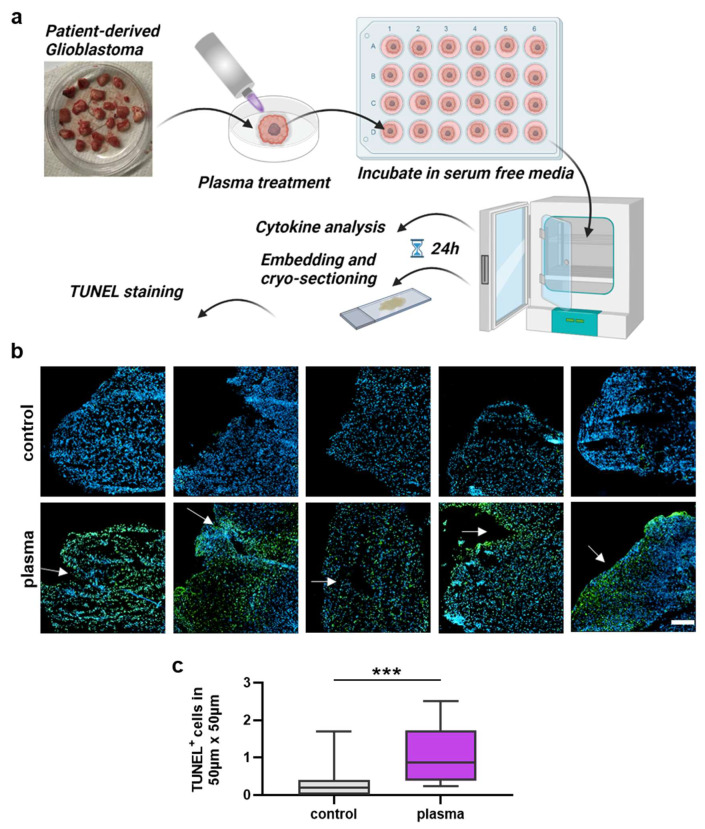
Patient-derived GBM tumor sample analysis. (**a**) Study protocol; (**b**) examples of highly apoptotic (TUNEL^+^, green) patient-derived glioblastoma multiforme tissue ultrathin cryosections following gas plasma exposure (nuclei counterstained with DAPI in blue); (**c**) algorithm-driven quantitative image analysis of tissue sections for TUNEL^+^ (apoptotic) cells per area. Data are box plots (Tukey) from 16 patient samples; statistical analysis was performed using two-tailed Mann–Whitney test with *p* < 0.001 (***) differing significantly. Scale bar is 150 µm.

**Figure 4:**
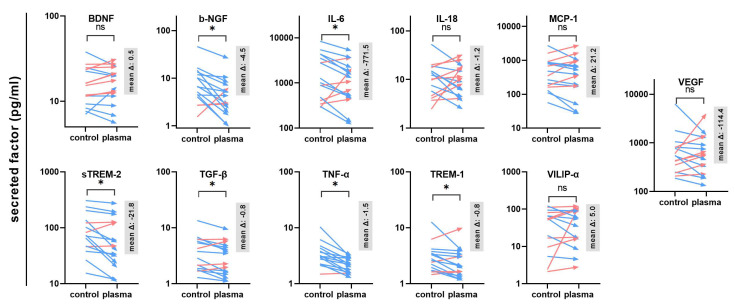
Secretion profiling of patient-derived GBM tissues. A total of 11 cytokines, chemokines, and growth factors were analyzed in the supernatants of untreated or gas plasma-treated patient-derived glioblastoma multiforme (GBM) tumor biopsies cultured for 24 h. Data are from 15–16 donors and show mean of each donor and control and treatment group (red: increase, blue: decrease) with mean delta (Δ) given in gray boxes; statistical analysis was performed based on normality distribution using either a paired *t*-test or two-tailed Wilcoxon rank test with *p* < 0.05 (*) differing significantly or non-significantly (ns).

The next question was whether gas plasma exposure affected patient-derived glioblastoma multiforme tissue biopsies ex vivo. Following the treatment, the tissues were cultured for 24 h before tissue section and secretion profile analysis (Figure 3a). TUNEL staining indicative of apoptotic cells was performed, and sections were imaged and analyzed by fluorescence microscopy and algorithm-driven quantitative image analysis. Gas plasma exposure led to strongly apoptotic regions in the GBM tissues when compared to untreated controls (Figure 3b). Quantitative analysis revealed a significantly greater presence of apoptotic cells in gas plasma-treated tissue than controls (Figure 3c). In the context of gas plasma treatment, ROS are not only known to increase cell death but also to modulate inflammation within the tumor. Hence, in the supernatants of these untreated and gas plasma-treated and cultured GBM tissue biopsies, an array of cytokines, chemokines, and growth factors was detected. The concentrations of several targets were significantly modulated by gas plasma exposure (Figure 4). TREM-1 (triggering receptor expressed on myeloid cells 1), BDNF (brain-derived neurotrophic factor), IL-18, TGF-β (tumor growth factor beta), TNF-α (tumor necrosis factor-alpha), and b-NGF (nerve growth factor beta) showed an overall low expression regardless of the treatment group. In turn, IL-6, VEGF (vascular endothelial growth factor), MCP-1, VILIP-α (visinin-like protein alpha/1), and sTREM-2 (soluble TREM2) were found to be released at modest to high levels. Ex vivo gas plasmas exposure of patient-derived primary glioblastoma tissue led to significantly decreased release levels of b-NGF, IL-6, sTREM2, TGF-β, TNF-α, and TREM-1.

## 4. Discussion

Gas plasma technology is a promising new avenue in cancer treatment. Recent reports have suggested a role of this treatment in glioblastoma therapy [28], but data on immunomodulatory consequences and translational models are scarce, which is addressed in the current study.

Upon gas plasma exposure, we found robust induction of apoptosis in patient-derived glioblastoma tissue treated ex vivo. The programmed cell death in glioblastoma is a major target for therapeutic strategies. Apoptosis is caspase-controlled and is divided into an extrinsic and intrinsic pathway. The extrinsic pathway is regulated by activating the receptors TNF-R1 or FAS DR4/DR5, which leads to the recruitment of caspases 8 and 10 with subsequent activation of effector caspases (caspase 3). In contrast, intracellular ROS increase, such as that which occurs with gas plasma exposure, activates the intrinsic pathway, which causes a conformational change in the pro-apoptotic BCL-2 proteins BAK and BAX with subsequent cytochrome c release and activation of caspase 3 via caspase 9. Apoptosis resistance in glioblastoma is a problematic aspect of glioblastoma control. In part, it indicates the poor response to radio and chemotherapy, which could be enhanced by the intervention of the resistance processes [29]. Specifically, in recurrent GBM, the pro-survival proteins BCL-2 and BCL-X_L_ are found to be upregulated, while the pro-apoptotic BAX is downregulated [30]. Until now, there has been a lack of strategy to increase apoptosis above the level of known established therapies [29]. An intracellular ROS increase, elicited via the addition of gas plasma-generated ROS, might be a putative way of inducing apoptosis, as has been previously shown in melanoma cells [31]. Intriguingly, we have also found gas plasma-mediated apoptosis induction in patient-derived metastatic melanoma lesions previously [32]. We recently extended these findings to other skin cancers, namely squamous cell carcinoma and basal cell carcinoma [33]. Additionally, primary-patient-derived breast cancer cells were demised in response to gas plasma treatment [34]. While controlled multipatient cancer studies using this technology are still awaited, these ex vivo results together with first case report collections [35] might motivate future clinical research on different tumor entities, including GBM. Promising in vivo findings in an orthotopic GBM mouse model by the group of Michael Keidar encourages research to continue on this path [10]. Strikingly, they also reported on the additive cytotoxic effects of gas plasma and Temozolomide treatment in vivo [11], a cytostatic drug used in GBM therapy. Another study found that in vitro gas plasma-treated U87 cells developed smaller tumors in vivo than untreated cells [36].

The tumor microenvironment (TME) in glioblastoma is known for its complexity. Besides apoptosis resistance, the TME composition is another reason for the lack of success of different anti-GBM therapies [37,38]. Cytokines, chemokines, and growth factors that significantly influence the development and growth of the tumor also play a part in this. Therefore, we have investigated 11 different cytokines in their expression, which often play a major role in neuro-inflammatory pathologies. IL-6, TGF-β, TNF-α, b-NGF, and sTREM-2 showed a significant reduction after gas plasma exposure. IL-6 has been shown to have a tumor-supportive effect. It promotes tumor growth and the migration of tumor cells in a similar fashion to a growth factor in glioblastoma [39,40]. In addition, IL-6 can block apoptosis and thus hamper therapeutic interventions [41]. Hence, a gas plasma-induced decreased release of IL-6 might sensitize GBM cells to apoptosis. The alternatively activated tumor-associated macrophages (TAM) are considered controllers of the TME and support cancer growth. It has been shown that IL-6 knock-out inhibits alternative macrophage activation and thus increased survival in a murine GBM model [42]. For TNF-α, evidence shows that this factor promotes anti-apoptotic effects [43]. Furthermore, TNF-α induces CCL2 (MCP-1), which is known for its immunosuppressive and pro-tumoral activity due to increased M2 (TAM) macrophage polarization [44]. In our study, however, the significantly decreased TNF-α upon the gas plasma treatment of GBM tissue did not correlate with significantly decreased MCP-1 levels. This might be due to differences in the cellular composition of the GBM tissue samples in terms of cells being the source of either factor, as MCP-1 can be released by both TAM and GBM cells [45,46]. In addition, tumor cells, including GBM, are known for their dysregulated signaling and epigenetic regulation, potentially resulting in TNF-α-independent MCP-1 release. TNF-α also promotes the JNK-Axl-ERK pathway [47,48], which is known to induce resistance to EGFR therapy, an important strategy in GBM treatment [49]. b-NGF binds to the high affinity Tropomyosin receptor Kinase A (TrKA), which leads to amplified VEGF and thus increased angiogenesis, migration [50], apoptosis inhibition via p75NTR [51], and enhanced proliferation through the Notch1 signaling pathway [52]. Along similar lines, TGF-β [53] and TREM-2 [54] elevated invasiveness and tumor growth in glioblastoma. TGF-β also activates matrix metalloproteinases [55,56] and promotes the polarization of cancer-supporting TAM [57,58]. Altogether, the gas plasma-mediated reduction in these factors plays a role not only in GBM cell death but also in the immunomodulation of the GBM TME. The effects of plasma treatment show important approaches to developing new therapeutic strategies.

Our in vitro findings are in line with previously published evidence. Increased intracellular ROS and a decline in metabolic activity was noted in U87 cells by Cheng and colleagues using a helium-driven plasma jet [59] as well as plasma-treated media [60]. Akter and colleagues have also observed an increase in the G1 to G2 ratio of the cell cycle following gas plasma-exposed glioblastoma cells [36], as observed in our study. The authors also found GBM cells to be more sensitive to gas plasma exposure than non-malignant astrocytes [61]. Albeit our comparison cell line HaCaT derives from the skin and is less suitable as the control, its greater sensitivity to the gas plasma treatment is in line with previous findings that some tumor cell types are more sensitive to this non-malignant cell type while others are less sensitive [62], exemplifying that such comparisons are often relative. What is striking, however, is the finding that the cell cycle arrest observed in our study was found to be independent of the gas plasma source type and condition, underlining the general ROS-based nature of this treatment modality [4]. The main finding in our monocyte–GBM co-culture was a decline in CD163 and CD169 surface marker expression and increased release of IL-8. CD163 is a marker of anti-inflammatory M2 macrophages [63] supporting tumor growth [64]. SIGLEC1 (CD169) is thought to characterize regulatory myeloid cells participating in immunological tolerance and erythropoiesis [65]. Recent reports, however, suggest CD169^+^ monocytes contribute to antiviral defense, including COVID-19 [66,67]. Either way, our results do not point to an increased tumor-promoting phenotype, while the opposite cannot be concluded either as we did not investigate other markers of monocyte-to-macrophage differentiation and polarization. As microglia (macrophages) are an often-dominating immune cell type in the GBM TME, our experiments might be extended to monocyte-derived macrophages to resemble this situation closer in future studies. Regarding IL-8, there is clear evidence of its detrimental role in GBM pathogenesis and disease recurrence [68,69]. However, THP-1 monocytes were previously found to increase IL-8 expression and be released after gas plasma-induced intracellular ROS increase independently of the presence of tumor cells [70], which might have been the case in our study also.

Gas plasma technology generates multiple reactive species, which leads to ROS/RNS overload in target cells and tissues. According to Helmut Sies, oxidative stress is defined as a disturbance in the prooxidant–antioxidant balance in favor of the former [71]. At the time of gas plasma treatment, there is an immediate prooxidant surplus in the target cell and tissue, albeit the direct and dynamic measurement of such event, especially in tissues, remains technically challenging as of today. Cells, including GBM, have antioxidant enzymes to counteract ROS/RNS overload. Glutathione peroxidase 1 is a prototypical example in GBM [72]. Moreover, it has been proposed that oxidative stress, induced by pharmacological or technological means, sensitizes GBM to radiotherapy [73]. This concept has recently been extended to fat catabolism, fatty-acid-induced mitochondrial dysfunction, and oxidative stress with toxic consequences in GBM cells [74]. Additionally, sodium sulfide has been reported as a potential radiosensitizer in GBM [75]. However, combination treatments of gas plasma and ionizing radiation have so far only been tested for melanoma and breast cancer but not glioblastoma cells [76,77].

Some glioblastomas are inoperable due to their location in the brain because of the danger of damaging brain tissue which is vital to life or because of tumors being located at major brain blood vessels [78]. There are particularly critical localizations near the speech center (left temporal or mesencephalic tumors near the brainstem). Hence, gas plasma technology could be a strategy to mediate cytotoxic and immunomodulatory effects locally to GBM tissue, especially in areas not amenable to surgical resection such as the brainstem or other eloquent brain areas. In animal experiments, attempts have already been made to treat such non-resectable areas locally with chemotherapeutic drugs using cannulas [79]. However, locally applied chemotherapeutic agents can cause severe side effects and damage to the functionally important areas, whereas local plasma treatment has so far been void of any severe side effects in exposed human tissues.

## 5. Conclusions

Our proof-of-concept study provides evidence of a cytotoxic as well as immunomodulatory role of gas plasma exposure in glioblastoma in vitro and in patient-derived GBM tissue ex vivo. Hence, this technology might contribute to developing new therapeutic strategies in GBM management, especially as adjuvant treatment or in non-resectable areas.

## Data Availability

Data are available from the corresponding author upon reasonable request.
